# Supplementary figures and images for: EMT Inducers Catalyze Malignant Transformation of Mammary Epithelial Cells and Drive Tumorigenesis towards Claudin-Low Tumors in Transgenic Mice
Source: PLoS Genet. 2012 May 24;8(5):e1002723. doi: 10.1371/journal.pgen.1002723 (PMC3359981; doi:10.1371/journal.pgen.1002723)

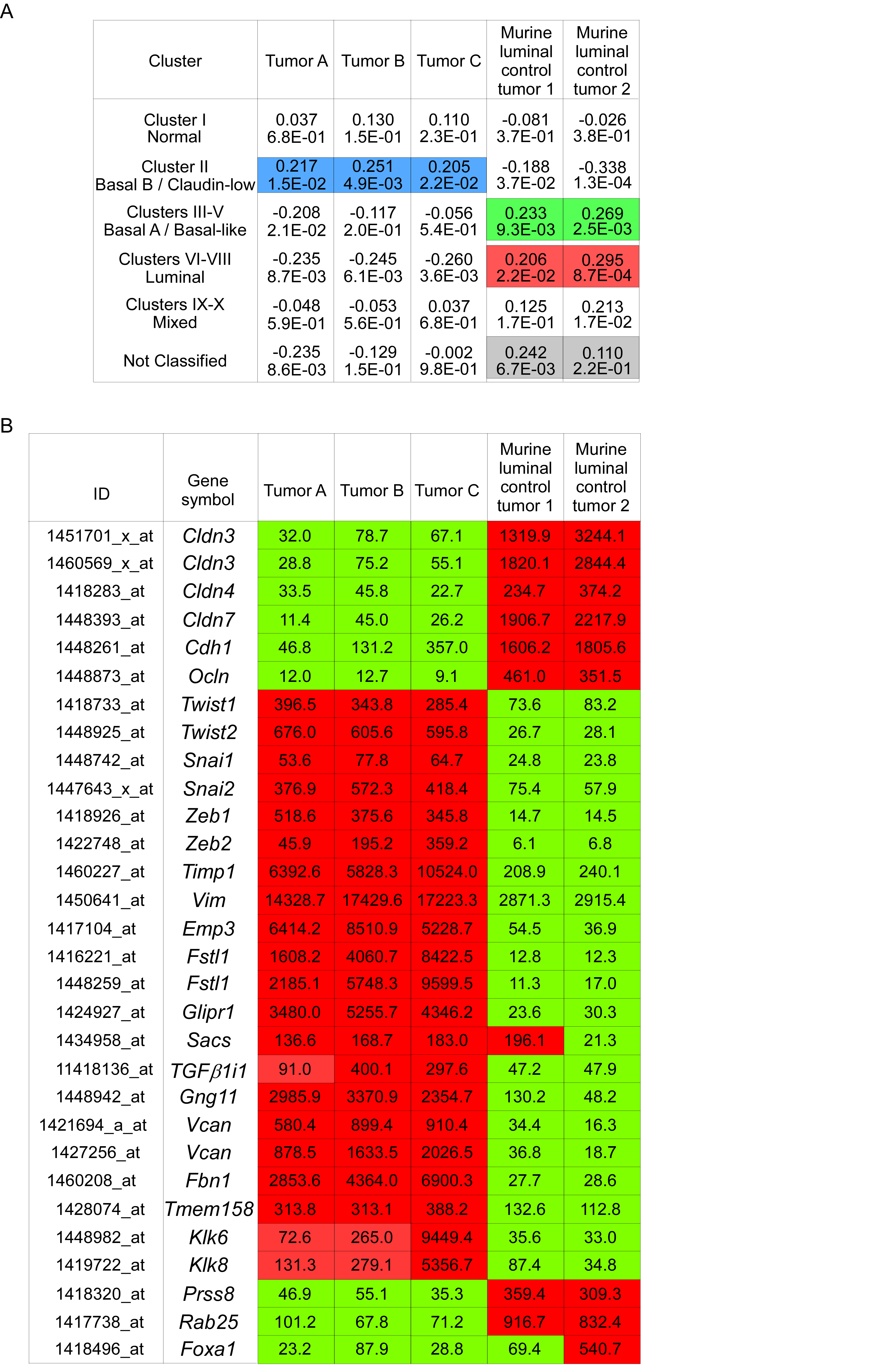

Supplement: Figure S1 — Mammary tumors developed by WAP-Cre;K-rasG12D;Twist1 mice exhibit a claudin-low gene expression signature. Gene expression profiles of WAP-Cre;K-rasG12D;Twist1-mouse derived tumors (A, B and C) and of two MMTV-Neu tumor-derived murine luminal cell lines were compared. (A) Statistical values of the Pearson's correlation to the centroids of intrinsic gene clusters are determined according to the 122 reference murine tumors ([13]). MMTV-Neu tumor-derived luminal cell lines were used as control luminal cells. (B) The expression of meaningful genes from the claudin-low signature ([13], [21]) is shown. Relative transcript abundance with highest expression is labeled in red and with lowest expression is labeled in green, respectively. (TIF) [file pgen.1002723.s001.tif]

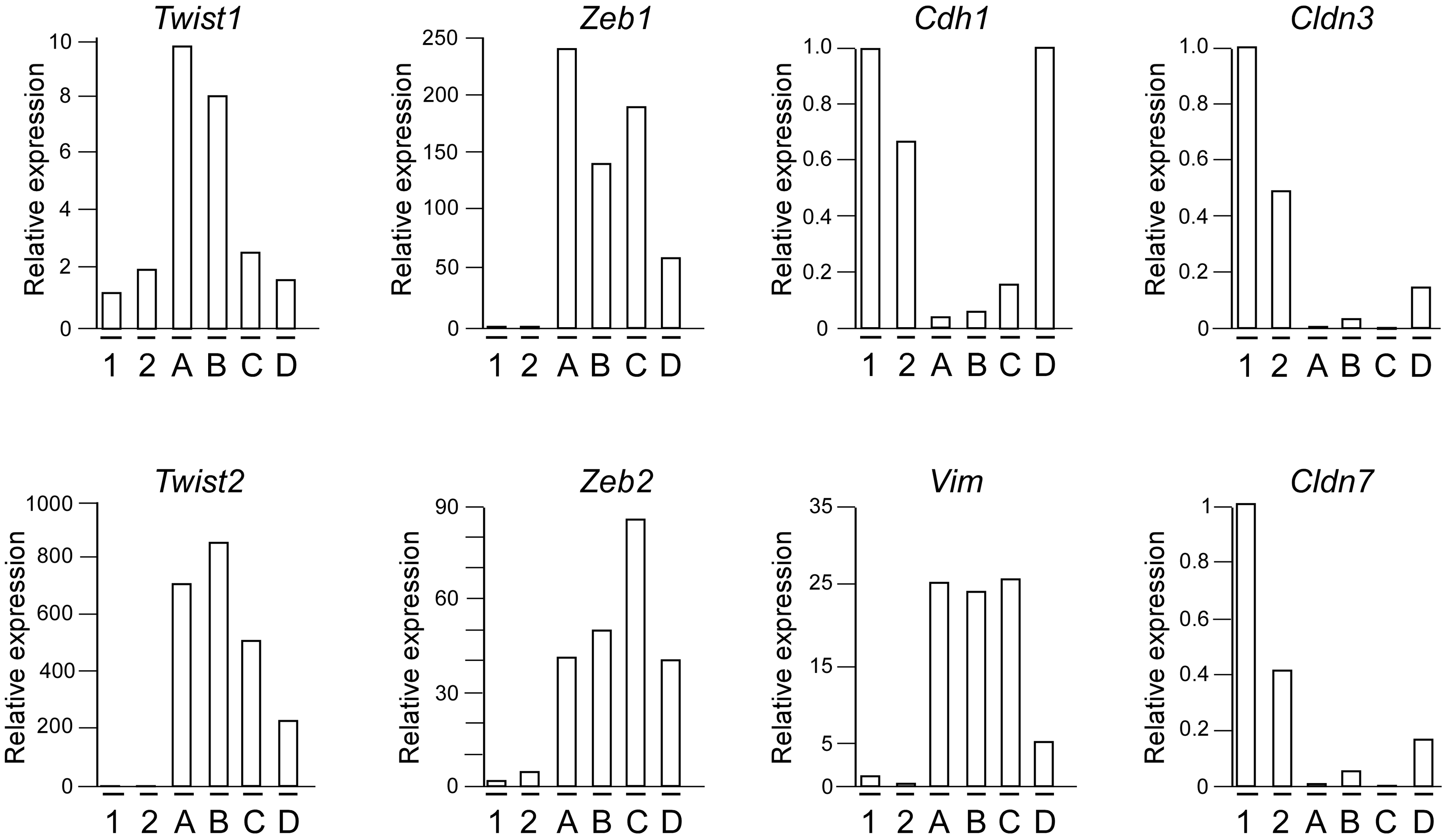

Supplement: Figure S2 — Characterization of the RAS+TWIST1 transgenic mouse-derived tumors. Expression analysis of Twist1/2, Zeb1/2, Cdh1, Vim, Cdln3 and Cdln7 in murine luminal control tumors 1 and 2 and the WAP-Cre;K-rasG12D;Twist1 (named RAS+TWIST) trangenic mouse derived tumors A to D, as assessed by Q-RT-PCR. Gene expression was assessed using the Hprt1 housekeeping gene as an internal control. The expression level was normalized with respect to murine luminal control tumor 1. (TIF) [file pgen.1002723.s002.tif]

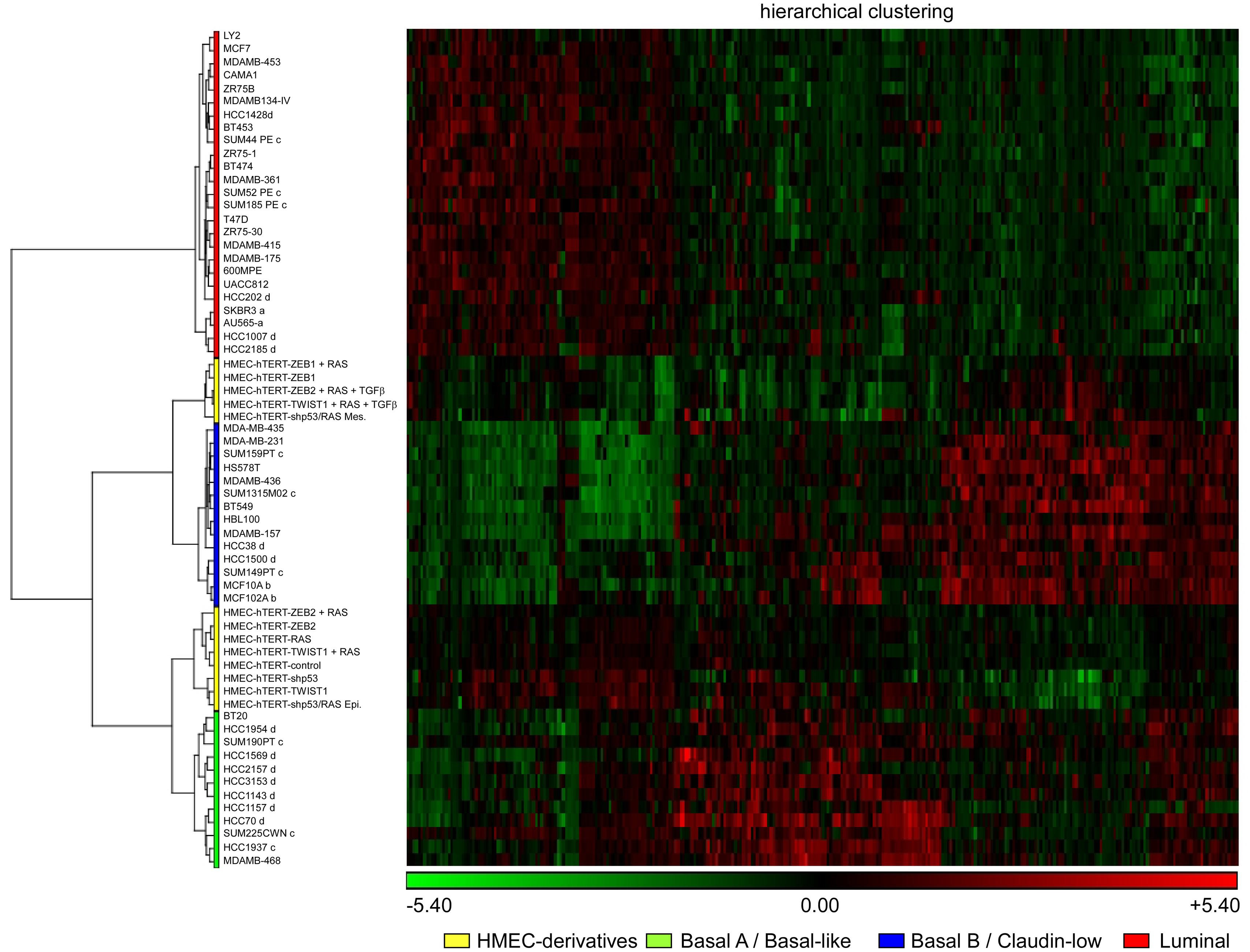

Supplement: Figure S3 — Combined expression of H-RASG12V and EMT-inducing transcription factors provides HMEC cells with a claudin-low gene expression signature. Hierarchical cluster analysis of the established HMEC-derived cell lines using the intrinsic gene clusters determined according to the cell line gene expression data set of Neve and collaborators [21]. The basal A/basal-like cluster is labeled in green, the basal B/claudin-low cluster is labeled in blue and the luminal cluster is labeled in red, HMEC-derived cell lines are labeled in yellow. (TIF) [file pgen.1002723.s003.tif]

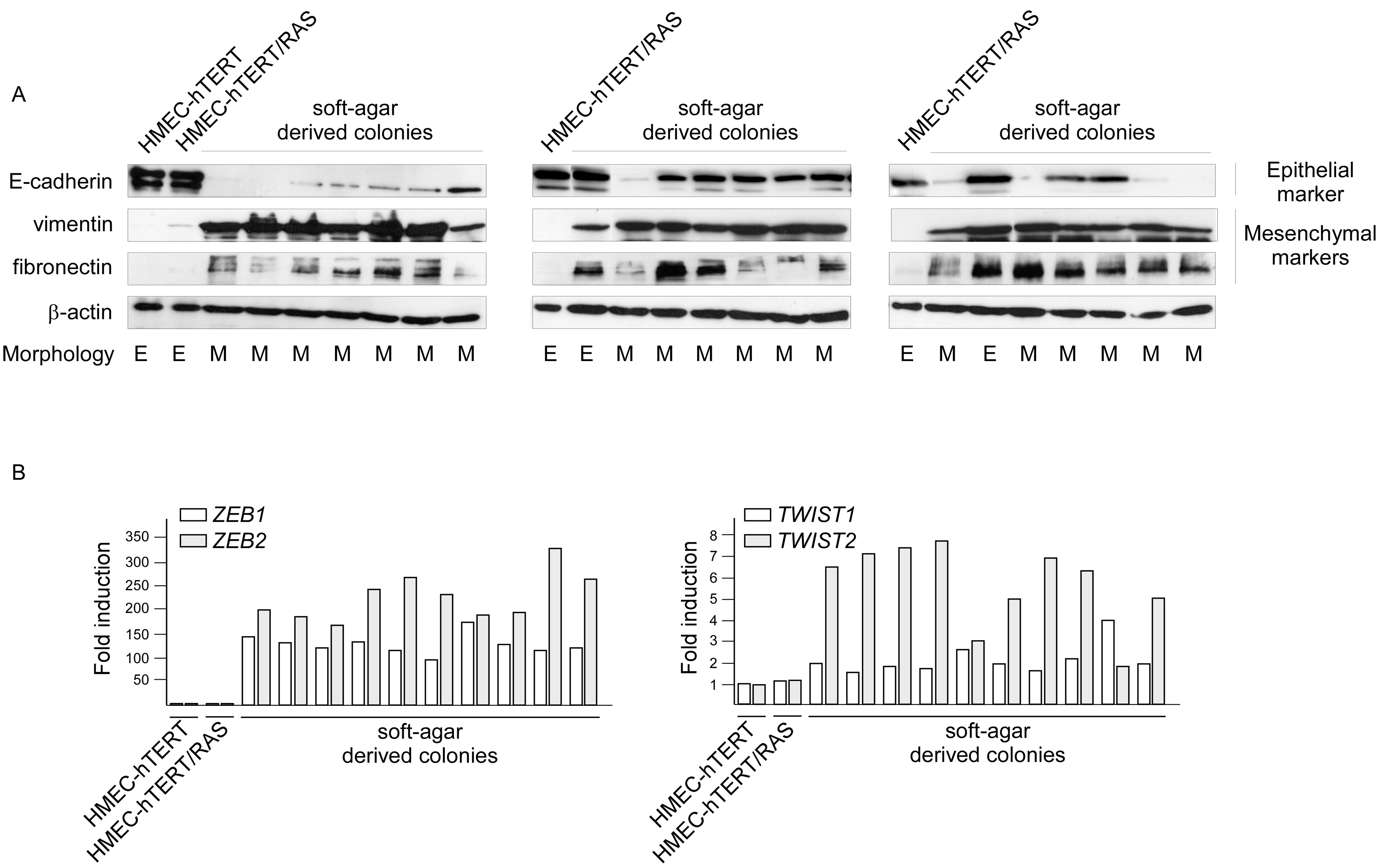

Supplement: Figure S4 — Transformed hTERT/RAS HMEC cells display EMT features. (A) Expression analysis of epithelial and mesenchymal markers in HMEC-hTERT cells, in HMEC-hTERT cells transduced with H-RASG12V (HMEC-hTERT/RAS) and in 21 independent HMEC-hTERT/RAS transformed colonies obtained in a soft-agar transformation assay. (B) mRNA expression analysis of EMT-inducing transcription factors (ZEB1, ZEB2, TWIST1 and TWIST2) as assessed by Q-RT-PCR in the ten colonies still expressing a significant level of E-cadherin. EMT-inducing transcription factor expression was assessed using the HPRT1 housekeeping gene as an internal control. The expression level was normalized with respect to HMEC-hTERT cells. E: Epithelial, M: Mesenchymal. (TIF) [file pgen.1002723.s004.tif]

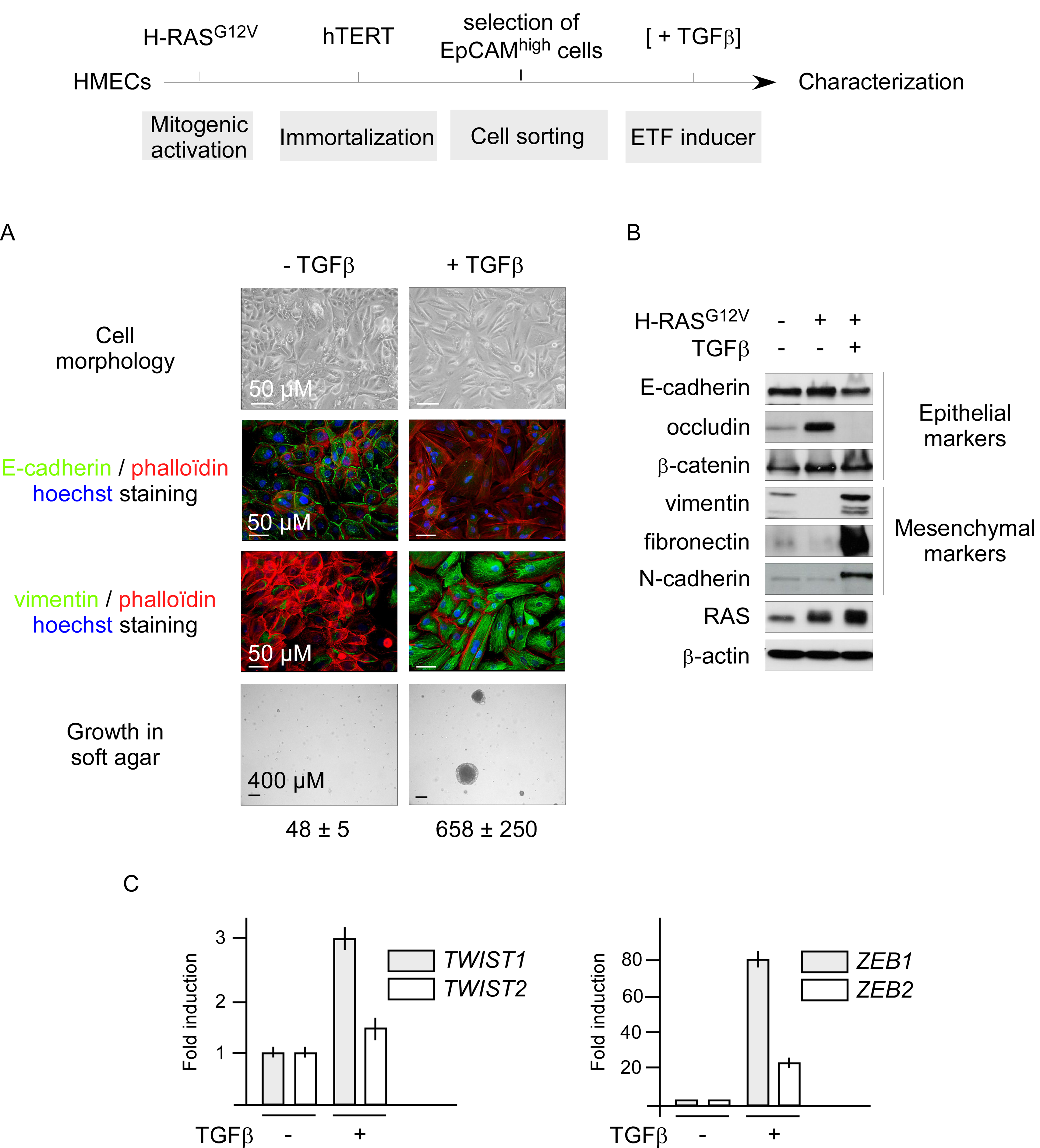

Supplement: Figure S5 — The EMT-promoting cytokine TGFβ cooperates with H-RAS for malignant transformation of HMEC cells. As depicted on top, HMEC cells were sequentially infected with H-RASG12V and immortalized with hTERT. The EpCAM+ epithelial cell population was sorted out and treated with TGFβ (2.5 ng/ml) for a three weeks period. EpCAM− mesenchymal cells were sorted out. The properties of the epithelial and mesenchymal isogenic cell lines were next compared. The optional step is indicated with brackets. (A) Upper panels: representative photomicrographs of cells obtained by phase-contrast microscopy. Middle panels: E-cadherin and vimentin expression analysis assessed by immunofluorescence. Lower panels: soft agar colony formation assay. Numbers of colonies are indicated ± SD of three replicates. (B) Expression analysis of epithelial and mesenchymal markers by western blotting. (C) TWIST1/2 and ZEB1/2 endogenous expression as assessed by Q-RT-PCR using the HPRT1 housekeeping gene as an internal control. The expression level was normalized with respect to the EpCAM+ sorted out epithelial cells. (TIF) [file pgen.1002723.s005.tif]

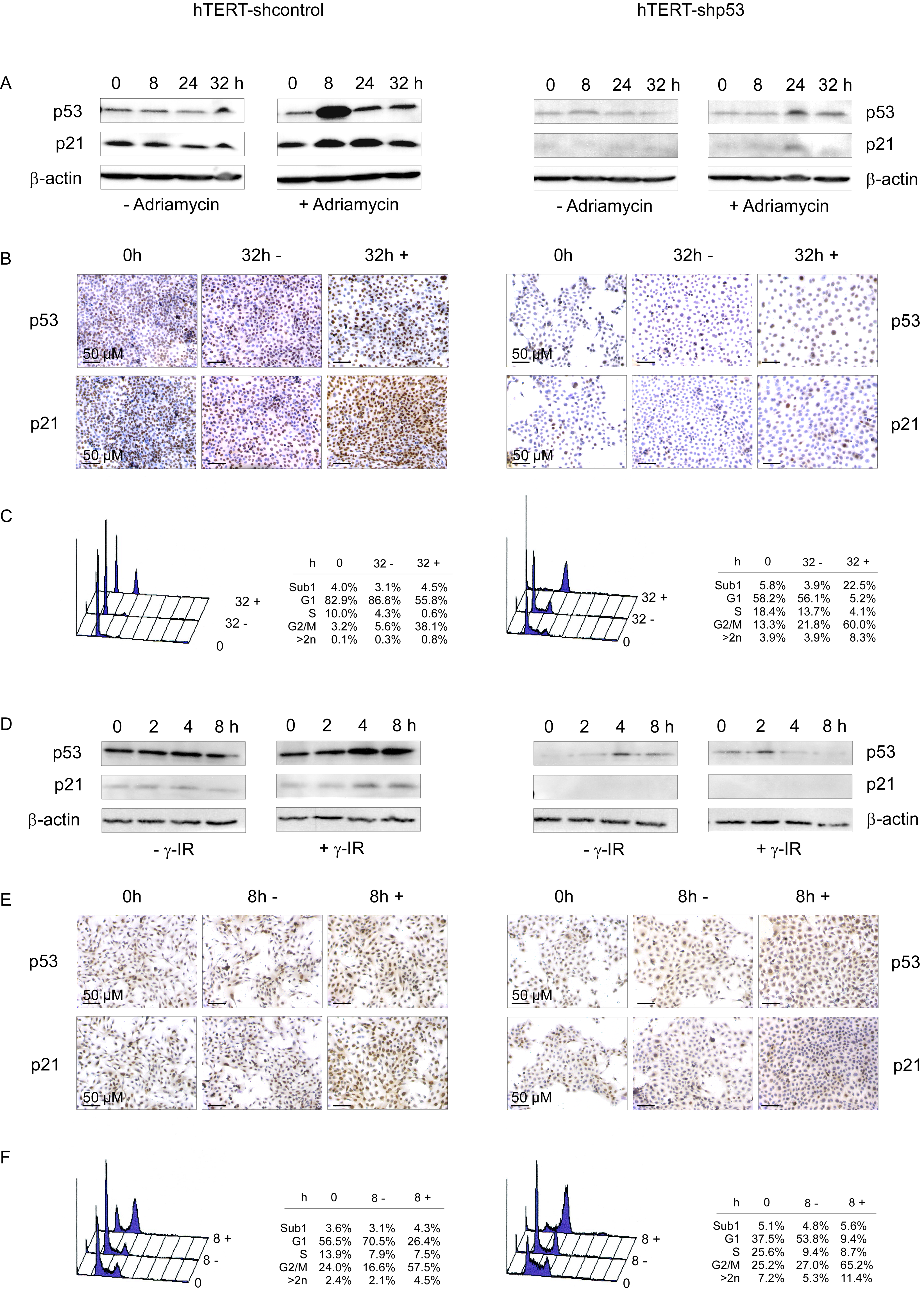

Supplement: Figure S6 — Confirmation of p53 pathway inactivation in p53-depleted cells. Expression analysis of p53, as assessed by western blotting (panels A and D) and immunohistochemistry (panels B and E), in hTERT-shcontrol (shRNA scramble) and hTERT-shp53 HMEC derivatives in response to DNA damage induction by adriamycin (+Adriamycin, panels A to C) or after γ-ray ionizing radiation (γ-IR, panels D to F). Expression in non-treated cells (−Adriamycin or - γ-IR) were used as controls. (Panels C and F) Cell-cycle distribution analysis before treatment (0 h) or 8 h/32 h post-treatment (8 h+, 32 h+). Cell cycle distribution in absence of treatment (8 h−, 32 h−) is shown. Percentages of cells distributed in the different cell cycle phases are indicated. (TIF) [file pgen.1002723.s006.tif]

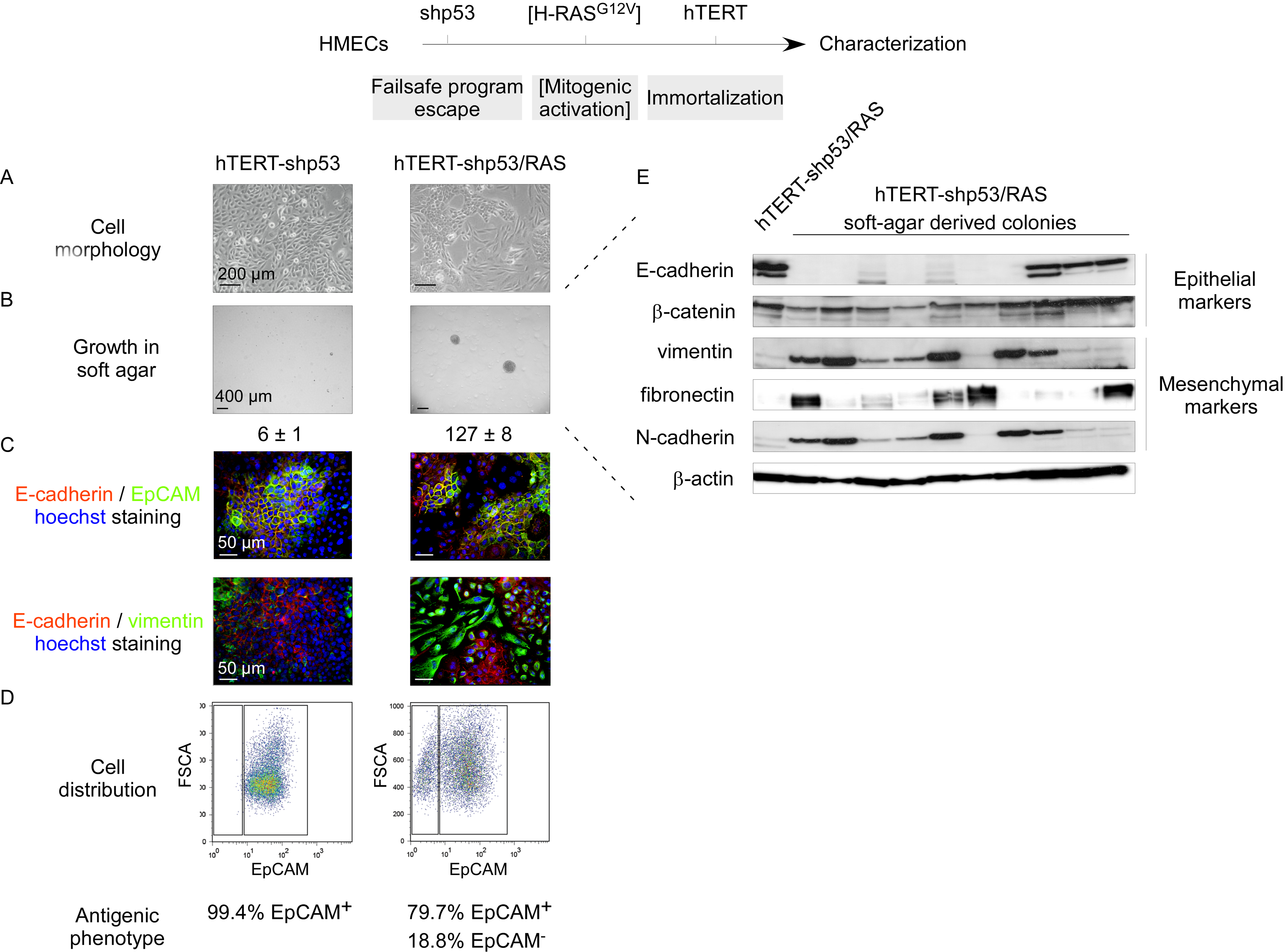

Supplement: Figure S7 — In vitro transformation assay of primary human mammary epithelial cells. HMEC cells were sequentially depleted of p53 through RNA interference (shp53), infected or not with H-RASG12V and immortalized by hTERT as depicted on top. The optional step is indicated by brackets. (A) Representative photomicrographs of cells obtained by phase contrast microscopy. (B) Transformation potential analysis, assessed by a soft agar colony formation assay. Numbers of colonies are indicated ± SD of triplicate experiments. (C) E-cadherin (TRITC), EpCAM (FITC) and vimentin (FITC) expression analysis assessed by immunofluorescence. (D) Cell distribution and antigenic phenotype analysis using the EpCAM antigen. (E) EMT marker expression analysis in transformed colonies as assessed by western blotting. (TIF) [file pgen.1002723.s007.tif]

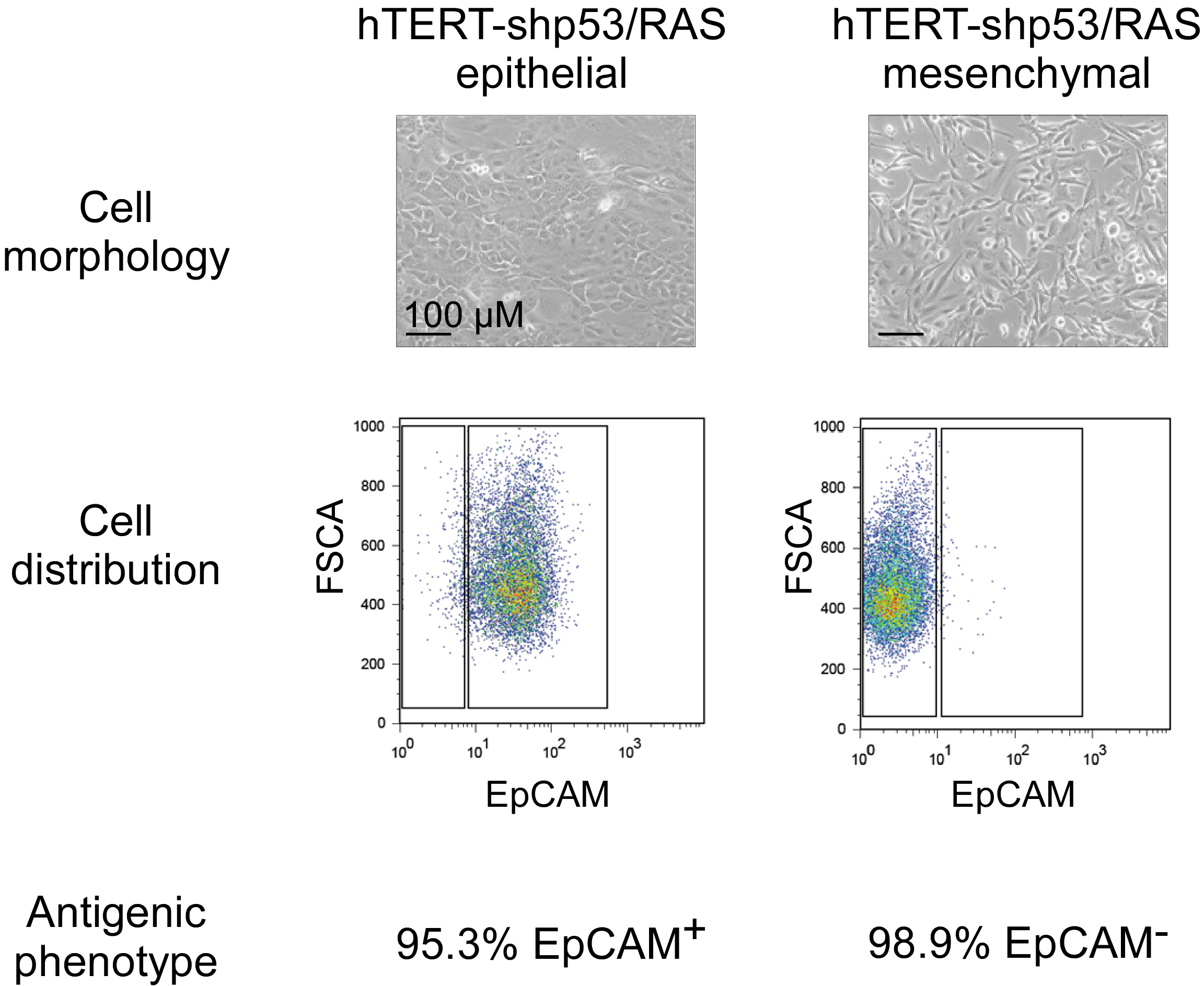

Supplement: Figure S8 — Separation of the epithelial and mesenchymal subpopulations of the h-TERT-shp53/RAS HMEC derived subpopulations. Cell morphology, cell distribution and the antigenic EpCAM phenotype are shown. (TIF) [file pgen.1002723.s008.tif]

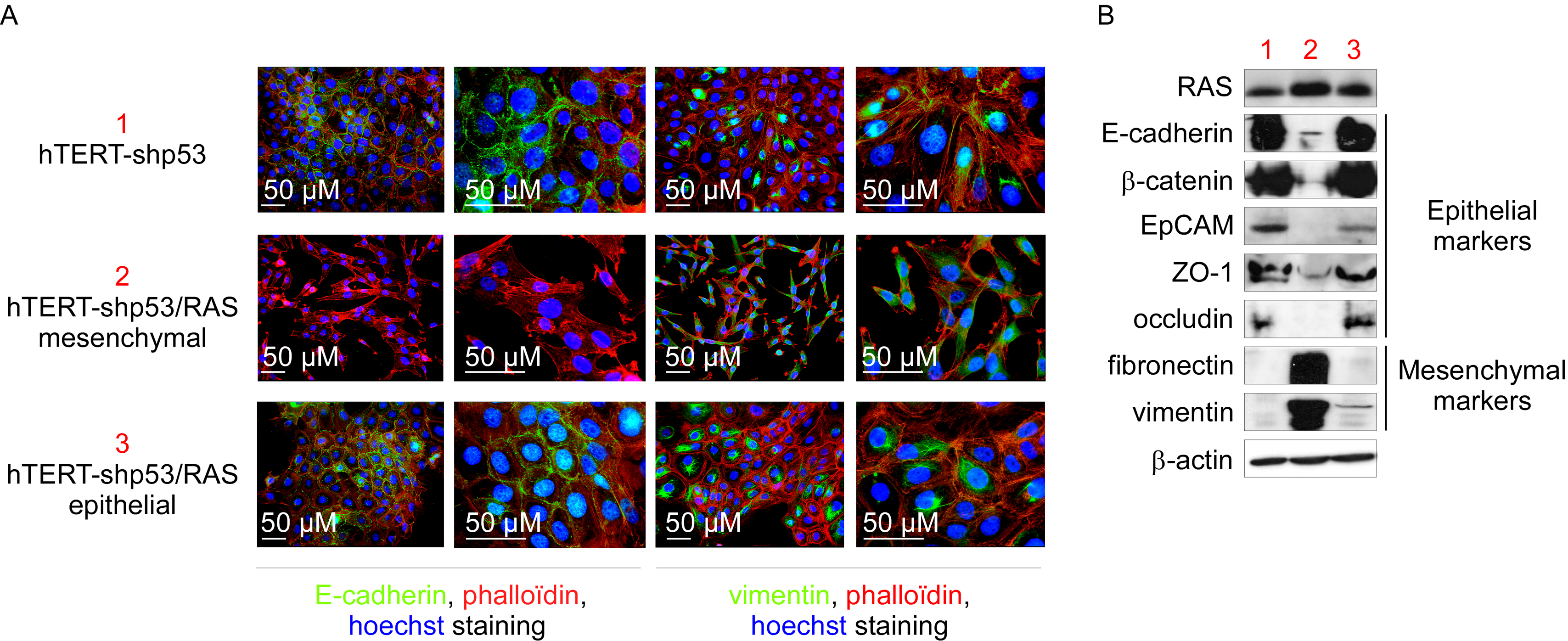

Supplement: Figure S9 — Characterization of the epithelial and mesenchymal hTERT-shp53/RAS HMEC derived subpopulations. (A) Expression analysis of the epithelial E-cadherin and the mesenchymal vimentin markers assessed by immunofluorescence in the sorted cell subpopulations, as indicated. (B) Expression analysis of epithelial and mesenchymal markers by western blotting. (TIF) [file pgen.1002723.s009.tif]

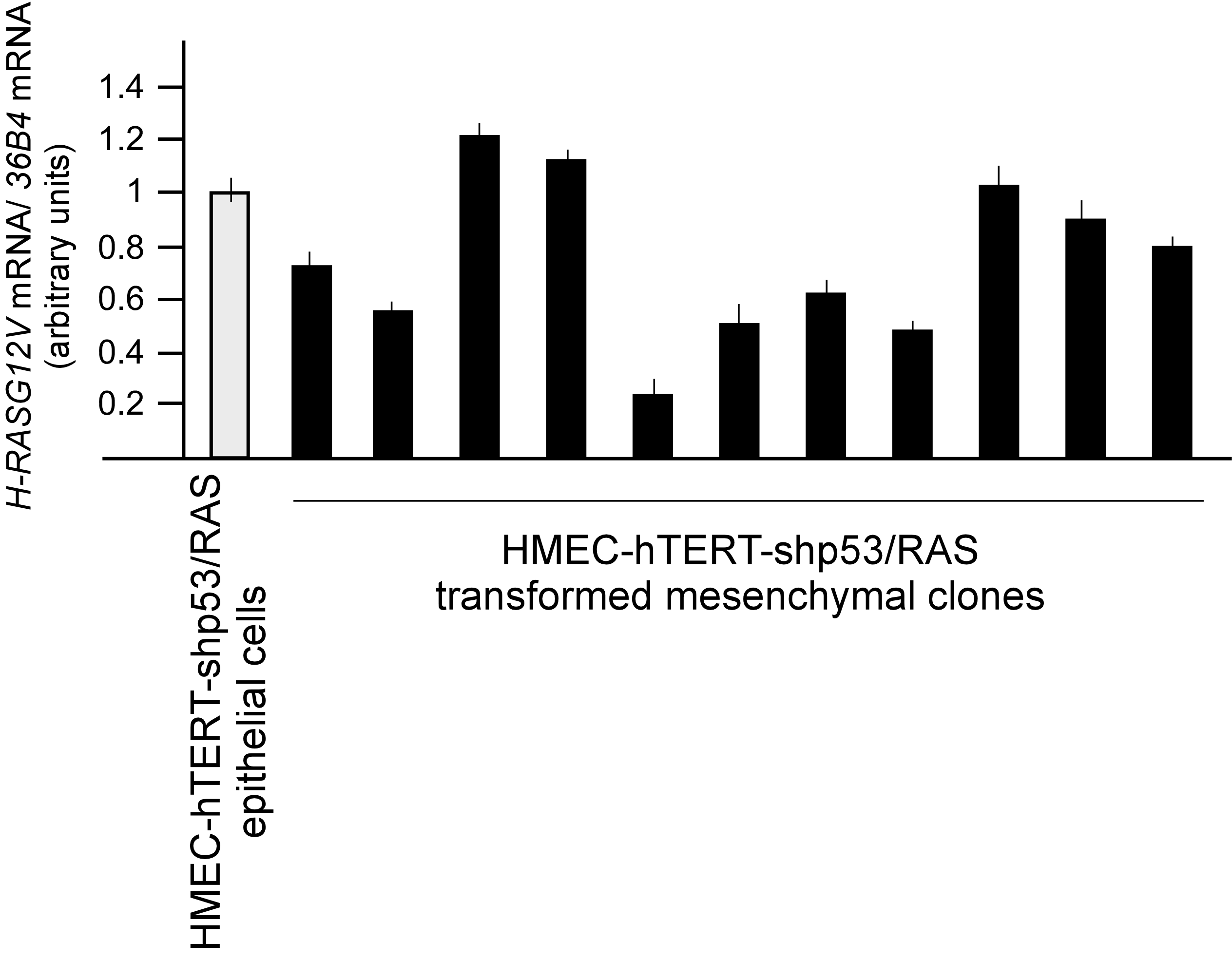

Supplement: Figure S10 — The differential oncogenic potential of mesenchymal and epithelial hTERT-shp53/RAS HMEC-derivatives does not rely on distinct H-RASG12V expression level. Ectopic expression of RAS in epithelial and transformed mesenchymal hTERT-shp53/RAS cells was assessed by Q-RT-PCR using the 36B4 gene as an internal control. The expression level was normalized with respect to hTERT-shp53/RAS epithelial cells. (TIF) [file pgen.1002723.s010.tif]

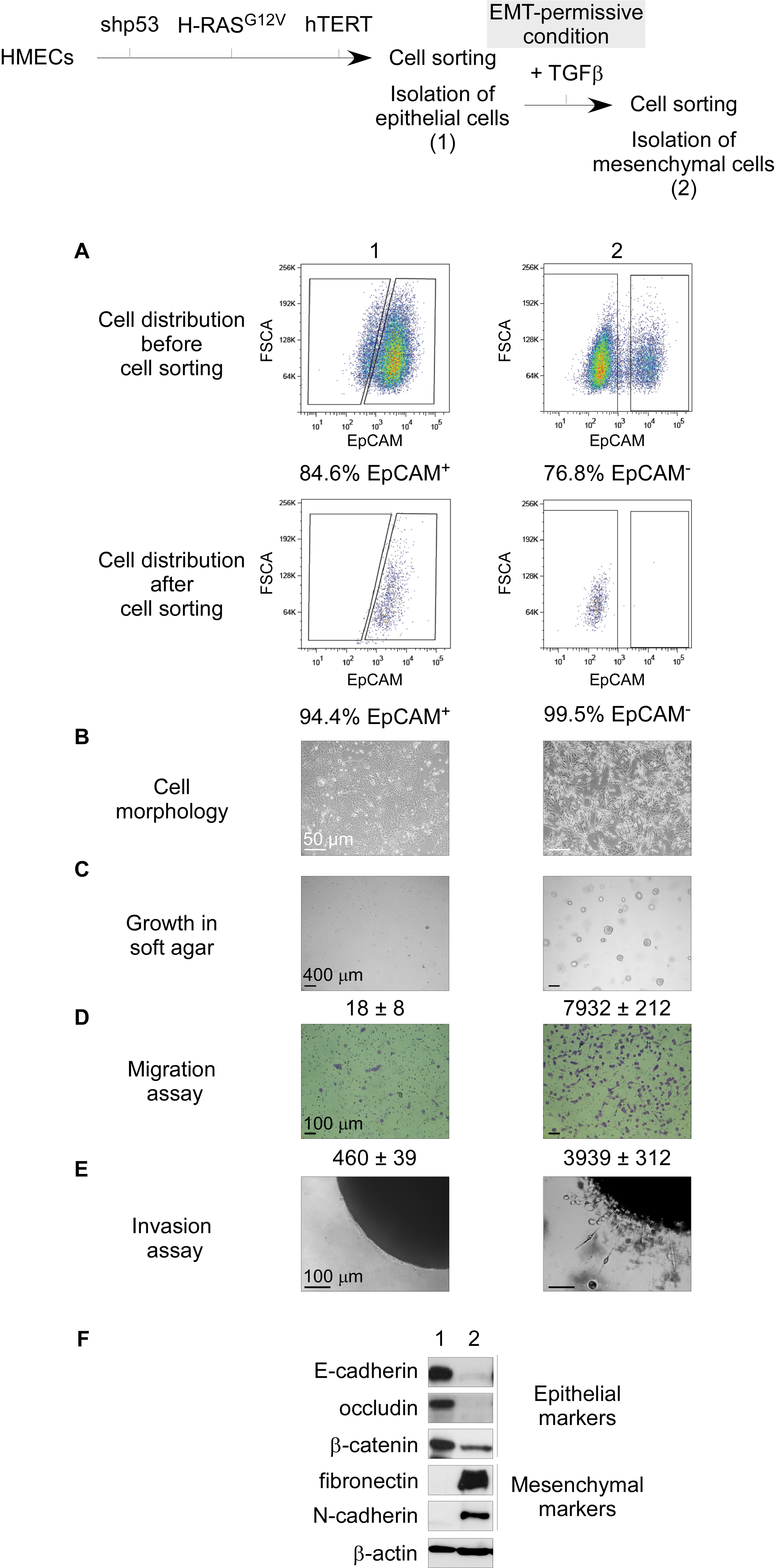

Supplement: Figure S11 — TGFβ-driven EMT provides hTERT-shp53/RAS HMEC cells with transformation potential, motility and invasive properties. Sequentially, HMEC cells were depleted in p53 (shp53), infected with H-RASG12V, and immortalized with hTERT. The epithelial subpopulation (1) was sorted out by FACS (EpCAM+ subpopulation) and treated with TGFβ (2.5 ng/ml) for a three weeks period. The resulting mesenchymal population (2) was sorted out by FACS (EpCAM− subpopulation). Experimental steps are schematized on top. Characterization of the epithelial and mesenchymal h-TERT-shp53/RAS isogenic cell lines. (A) Cell distribution before and after cell sorting. Percentages of EpCAM+ or EpCAM− cells are indicated. (B) Representative photomicrographs of cells obtained by phase-contrast microscopy. (C) Soft agar colony formation assay. Numbers of colonies are indicated ± SD of triplicate experiments. (D, E) Migratory and invasive properties analyses as assessed by Boyden chamber migration and Matrigel invasion assays, respectively. (F) Expression analysis of epithelial and mesenchymal markers by western blotting. (TIF) [file pgen.1002723.s011.tif]

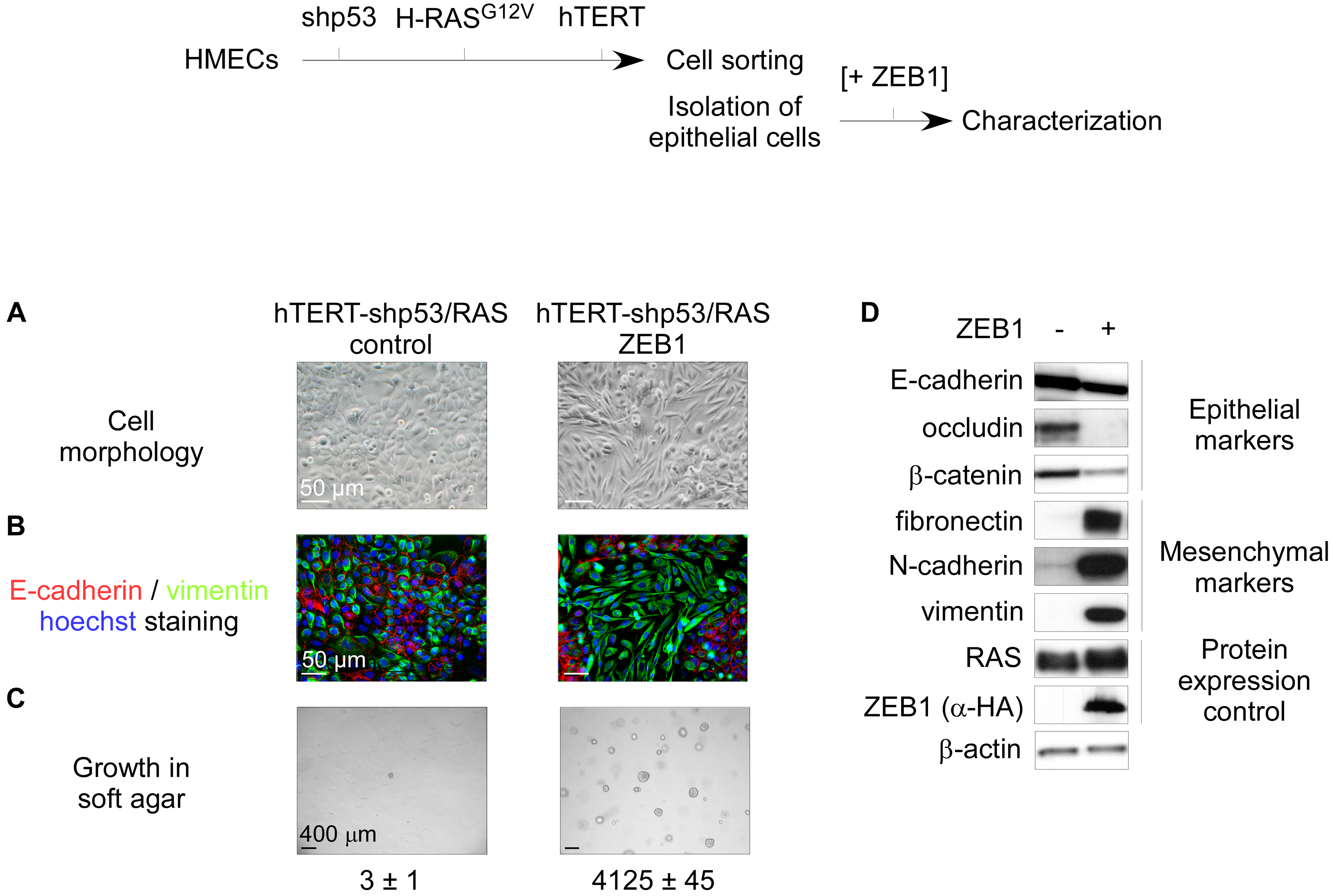

Supplement: Figure S12 — Transition from p53-depleted H-RASG12V-expressing epithelial cells into mesenchymal cells following ectopic expression of ZEB1 provides cells with a transformation potential. Ectopic expression of ZEB1 in sorted epithelial hTERT-shp53/RAS HMEC cells induced cell commitment to EMT, as indicated by the morphological change (A) the assessment of E-cadherin and vimentin expression by immunofluorescence (B) and the assessment of EMT marker expression by western blotting (D), provides cells with a transformation potential as assessed in a soft-agar colony assay (C). (TIF) [file pgen.1002723.s012.tif]

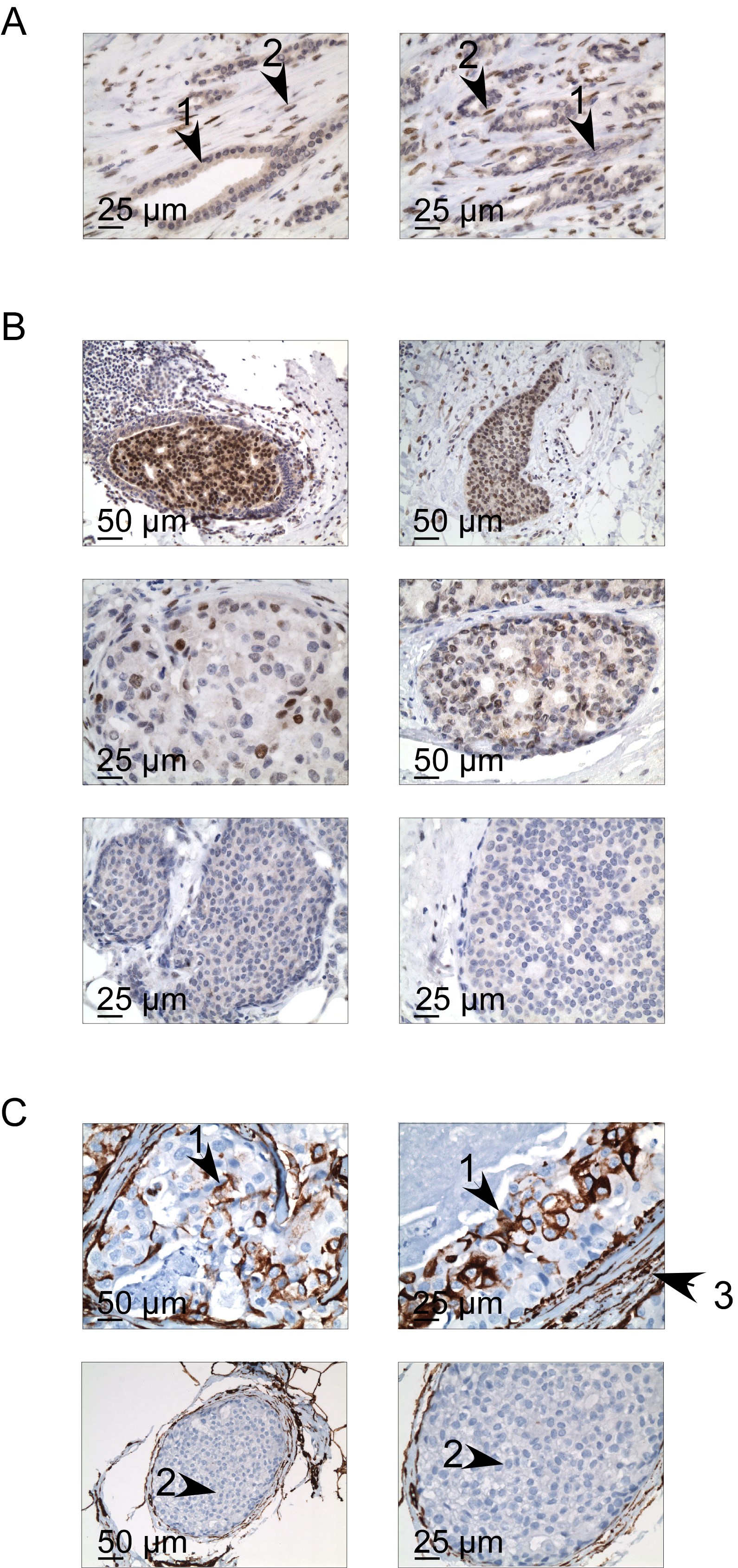

Supplement: Figure S13 — TWIST1 expression in human breast in situ carcinomas. (A) Immunohistochemical analysis of TWIST1 expression in the normal mammary gland. While TWIST1 is not expressed in mammary epithelial cells (arrows 1), the protein is detected in some stromal fibroblasts (arrows 2). (B) TWIST1 expression was analyzed by immunohistochemistry in 34 human ductal carcinomas (DCIS) of the breast. Representative TWIST1 staining of independent samples is shown. TWIST1 protein was detected in 18 of them. Remarkably, in 11 of these cases TWIST1 was homogenously expressed in the bulk of the lesion even while cancer cells maintained an epithelial phenotype. Top panels: positive samples harbouring a strong and homogenous staining, Middle panels: positive samples harbouring a weak and/or heterogenous staining, Lower panels: negative samples. (C) Analysis of vimentin expression by immunohistochemistry in TWIST1-positive DCIS. Among the 18 TWIST1-positive samples, 6 expressed significant levels of vimentin while maintaining an epithelial phenotype. Arrows 1: vimentin positive epithelial cells. Arrows 2: vimentin negative epithelial cells. Arrow 3: normal myoepithelial cells expressing vimentin. (TIF) [file pgen.1002723.s013.tif]
