# Supplementary material for: EMT Inducers Catalyze Malignant Transformation of Mammary Epithelial Cells and Drive Tumorigenesis towards Claudin-Low Tumors in Transgenic Mice
Source: PLoS Genet. 2012 May 24;8(5):e1002723. doi: 10.1371/journal.pgen.1002723 (PMC3359981; doi:10.1371/journal.pgen.1002723)
Supplement: Text S1 — Supplementary Materials and Methods. Text S1 includes details of procedures employed to perform the transcriptional expression analysis, the microarray processing and analysis, the analysis of the p53 pathway functionality, the flow cytometry analysis, the immunochemistry on human samples and the assessment of the ectopic H-RASG12V expression. (DOC) [file pgen.1002723.s014.doc]

**Supplementary Information**

**EMT inducers catalyze malignant transformation of mammary epithelial cells and drive tumorigenesis towards claudin-low tumors in transgenic mice**

Anne-Pierre Morel1,2,3,4,5,6^†^, George W. Hinkal1,2,3,4,5,6^†^, Clémence Thomas1,2,3,4,5,6^†^, Frédérique Fauvet1,2,3,4,5,6, Stéphanie Courtois-Cox1,2,3,4,5,6, Anne Wierinckx1,2,3,4,5,7, Mojgan Devouassoux-Shisheboran1,2,3,4,5,8, Isabelle Treilleux1,2,4,6, Agnès Tissier1,2,3,4,5,6, Baptiste Gras1,2,3,4,5,6, Julie Pourchet1,2,3,4,5,6, Isabelle Puisieux1,2,3,4,5,6, Gareth J. Browne9, Douglas B. Spicer10, Joël Lachuer1,2,3,4,5,7, Stéphane Ansieau1,2,3,4,5,6* and Alain Puisieux1,2,3,4,5,6,11^¶^

**Materials and Methods**

*Transcriptional expression analysis.*

TaqMan quantitative PCR analysis was carried out on a LightCycler 2.0 System (Roche Applied Science). Real-time PCR intron-spanning assays were designed using the ProbeFinder software (Roche Applied Science). Sequences of primers and probes are listed below:

Human *TWIST1*: GGCTCAGCTACGCCTTCTC and CCTTCTCTGGAAACAATGACATCT (probe 88), human *TWIST2*: CATGTCCGCCTCCCACTA and GCATCA-TTCAGAATCTCCTCC (probe 10), human *ZEB1* AACTGCTGGGAGGATGACA and TCCTGCTTCATCTGCCTGA (probe 57), human *ZEB2* AAGCCAGGG-ACAGATCAGC and GCCACACTCTGTGCATTTGA (probe 68), human *HPRT1* TGACCTTGATTTATTTTGCATACC and CGAGCAAGACGTTCAGTCCT (probe 73), murine *Twist1* agctacgccttctccgtct and tccttctctggaaacaatgaca (probe 58), murine *Twist2* agctacgccttctccgtct and gatgtgcaggtgggtcct (probe 10), murine *Zeb1* gccagcagtcatgatgaaaa and tatcacaatacgggcaggtg (probe 48), murine *Zeb2* ccagaggaaacaaggatttcag and aggcctgacatgtagtcttgtg (probe 42), murine *Vim1* ccaaccttttcttccctgaa and tgagtgggtgtcaaccagag (probe 109), murine *Cdh1* atcctcgccctgctgatt and accaccgttctcctccgta (probe 18), murine *Cldn3* tgggagctgggttgtacg and caggagcaacacagcaagg (probe 26), murine *Cldn7* gacgcccatgaacgttaagta and cctggacaggagcaagagag (probe 93), and murine *Hprt1* tcctcctcagaccgctttt and cctggttcatcatcgctaatc (probe 95).

*Microarray processing*

Microarray processing and data analysis were performed on the ProfileXpert core facility (Bron, France). Total RNA (100 ng) was amplified and biotin-labeled using Kit GeneChip 3’ IVT Express and procedures from Affymetrix (Santa Clara, CA, USA). Microarrays analyses were performed using high-density oligonucleotide arrays (Human Genome U133 Plus 2 or Mouse Genome 430 2.0 Array, Affymetrix, Santa Clara, CA, USA). Fifteen μg of biotinylated cRNA were fragmented and hybridization on chip was performed following Affymetrix protocol http://www.affymetrix.com. Arrays were washed and stained with streptavidin-phycoerythrin (Invitrogen Corporation, CA, USA) in a Fluidics Station 450 (Affymetrix) according to the manufacturer's instructions. The arrays were scanned with a confocal laser (Genechip scanner 3000, Affymetrix).

*Microarray analysis*

CEL files were generated using the Affymetrix GeneChip Command Console (AGCC) software 3.0. The complete set of CEL files is available at the GEO database under accession number GSE32905. The obtained data were normalized with Affymetrix Expression Console software using Robust Multiarray Average (RMA) statistical algorithm. Probesets were median-centered using Partek Genomic Suite software 6.6 (Partek Inc., St. Louis, MO, US).

In order to classify the HMEC-derivatives, we first used the median centered normalisation for all our human samples and the 52 reference cell lines published by Neve et al [1]. To validate the normalisation process between our samples and the 52 reference cell lines published [1] was added the MCF7, MDAMB157 as common references between the two sets of data by unsupervised clustering (data not shown). We then classified the parental HMEC-hTERT cell line and the HMEC-hTERT-RAS, HMEC-hTERT-TWIST1, HMEC-hTERT-ZEB1, HMEC-hTERT-ZEB2, HMEC-hTERT-RAS + TWIST1, HMEC-hTERT-RAS + ZEB1, HMEC-hTERT-RAS + ZEB2, HMEC-hTERT-RAS + TWIST1 with TGFβ, HMEC-hTERT-RAS + ZEB2 with TGFβ, HMEC-hTERT-shp53, HMEC-hTERT-shp53/RAS Epi and HMEC-hTERT-shp53/RAS Mes cell lines and the 52 reference cell lines using a previously established signature [2].

Clustering was performed using the Pearson’s dissimilarity algorithm and visualized using Partek Genomic Suite software 6.6. Moreover, Pearson’s correlations were calculated between our three 20 cell lines and centroids obtained for each gene signatures Basal A, Basal B or Luminal with the 52 reference cell lines [2].

We used the three signatures MaSc (mammary stem cells), LumProg (Luminal Progenitor ) and LumMat (Luminal Mature) established by Lim and collaborators [25] to appreciate the differentiation state of parental HMEC-hTERT cell line and the HMEC-hTERT-RAS, HMEC-hTERT-TWIST1, HMEC-hTERT-ZEB1, HMEC-hTERT-ZEB2, HMEC-hTERT-RAS + TWIST1, HMEC-hTERT-RAS + ZEB1, HMEC-hTERT-RAS + ZEB2, HMEC-hTERT-RAS + TWIST1 with TGFβ, HMEC-hTERT-RAS + ZEB2 with TGFβ, HMEC-hTERT-shp53, HMEC-hTERT-shp53/RAS Epi. and HMEC-hTERT-shp53/RAS Mes. We calculate an index of differentiation using the genes of the three signatures and as references the data of Basal B, Basal A or Luminal [1] whose concordance with the subpopulations MaSC, LumProg and LumMat were respectively established by Prat et al [2,3]. Expression signature index are defined as weighted average as described by Lim et al [4].


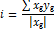


where the sum is over genes in the signature set, x_g_ is the average log2-fold-change for that gene in the cell lines references in concordance to the subpopulations and y_g_ is log2-expression for the same gene in our cell lines. Higher index indicate that the expression signature found in the reference cell lines is also found in our cell lines. In order, to compare the differentiation state of our cell lines, we used the calculated index describe above. Then, we divide our cell lines into two groups. The two cell groups represent epithelial (parental HMEC-hTERT , HMEC-hTERT-RAS, HMEC-hTERT-TWIST1, HMEC-hTERT-ZEB2, HMEC-hTERT-RAS + TWIST1, HMEC-hTERT-RAS + ZEB2, HMEC-hTERT-shp53 and HMEC-hTERT-shp53/RAS Epi.) and mesenchymal (HMEC-hTERT-ZEB1, HMEC-hTERT-RAS + ZEB1, HMEC-hTERT-RAS + TWIST1 with TGFβ, HMEC-hTERT-RAS + ZEB2 with TGFβ. and HMEC-hTERT-shp53/RAS Mes.) phenotypes. Unpaired T test was performed using GraphPad Prism version 5.04 for Windows (GraphPad Software, San Diego California USA, [www.graphpad.com](http://www.graphpad.com/)).

*Analysis of the p53 pathway functionality*

HMEC derivatives were either X-irradiated with 10 Gy or placed in presence of 200 nM Adriamycin®. After described periods of time, cell cycle profiles were determined using the FACs Cell Quest pro software and p53/p21^WAF1/CIP1^ expression was examined by western blotting and immunocytochemistry using monoclonal mouse anti-human p21^WAF1/CIP1^ clone SX118 and anti-human p53 clone DO-7 primary antibodies (Dako) and peroxidise anti-mouse antibodies (Dako), respectively.

*Flow Cytometry Analysis*

Trypsinized cells were washed in PBS, fixed in cold 70% ethanol/PBS for 30 min, washed, and incubated for 1 hr in 1 ml PBS containing 0.1 mg/ml RNase A and 20 mg/ml propidium iodide. Cell-cycle profiles were then determined using a FACSCalibur flow cytometer (BD Biosciences) and data were analyzed using ModFit software.

*Human tumor samples.*

Tissue specimens were obtained from Eric Tabone (Biological Resources Department, Centre Léon Bérard, French agreement number DC-2008-99). All human tissue samples were obtained after approval by the Comité de Protection des Personnes Lyon-Est and by the institutional review board and ethics committee of Centre Léon Bérard, with fully informed patient consent.

*Immunohistochemistry on human samples.*

Antigen retrieval was obtained by boiling the tissue sections in a pH 9 buffer using a PT Link (Dako). The slides were then incubated at 4°C overnight with the mouse monoclonal Twist2C1a anti-TWIST1 antibody (Abcam) or the polyclonal anti-vimentin SC7557 (Santa Cruz). After rinsing in Phosphate Buffer Saline, the slides were revealed with the Kit Envision FLEX, high pH (Dako) in the autostainer Link 48 (Dako).

*Assessment of the ectopic H-RASG12V expression*

The ectopic expression of *H-RASG12V* was assessed by SYBR green quantitative PCR by employing the primer pair TTCTCTAGGCGCCGGCCGGAT and GACGGCGCCAACAACAACAAGCTT located in 5’untranslated sequences provided by the pBabe retroviral vector and at the 5’ extremity of the *H-RAS* open reading frame. The *36B4* gene was used as an internal control, by employing the GCTGATGGGCAAGAACACCA and CCGGATGTGAGGCAGCA primer pair.

**References**

1. Neve RM, Chin K, Fridlyand J, Yeh J, Baehner FL, et al. (2006) A collection of breast cancer cell lines for the study of functionally distinct cancer subtypes. Cancer Cell 10: 515-527.

2. Herschkowitz JI, Simin K, Weigman VJ, Mikaelian I, Usary J, et al. (2007) Identification of conserved gene expression features between murine mammary carcinoma models and human breast tumors. Genome Biol 8: R76.

3. Prat A, Parker JS, Karginova O, Fan C, Livasy C, et al. (2010) Phenotypic and molecular characterization of the claudin-low intrinsic subtype of breast cancer. Breast Cancer Res 12: R68.

4. Lim E, Vaillant F, Wu D, Forrest NC, Pal B, et al. (2009) Aberrant luminal progenitors as the candidate target population for basal tumor development in BRCA1 mutation carriers. Nat Med 15: 907-913.
